# Changes in cardiorespiratory fitness and cardiovascular health in the workplace: a case study

**DOI:** 10.17159/2078-516X/2020/v32i1a7638

**Published:** 2020-01-01

**Authors:** G Torres, PJ Gradidge, D Constantinou

**Affiliations:** Centre for Exercise Science and Sports Medicine, Faculty of Health Sciences, University of the Witwatersrand, Johannesburg, South Africa

**Keywords:** cardiopulmonary fitness, exercise interventions, medical health claims, corporate wellness

## Abstract

**Background:**

Cardiorespiratory fitness (CRF) is an independent predictor of cardiovascular (CV) and all-cause mortality, contributing a higher proportion of CV risk compared to other traditionally recognised risk factors. However, CRF is not included in usual workplace wellness protocols and, as such, employers are not aware of the importance of this factor.

**Aim:**

The aim of this case study was to explore the effect of a 12-week exercise intervention programme on CRF, CV health and medical health claims in a male participant who was employed by a corporate company with existing chronic diseases.

**Findings:**

Health outcome measures improved after the 12-week exercise intervention programme. CRF showed the greatest improvement and medical health claims were lowered during the three-month post-intervention period.

**Implications:**

CRF should be included as a health outcome measure in worksite wellness programmes and monitored.

Workplace wellness programmes have been given increasing attention as a means to improve employee health and lower health costs ^[1]^. Most wellness programmes measure and report on cardiovascular (CV) risk factors, such as nutrition, smoking cessation, weight loss, and stress management. Cardiorespiratory fitness (CRF), an indicator of physical health-related fitness, has been identified as an independent predictor of CV and all-cause mortality and is also the risk factor with the highest percentage to all-cause deaths when compared to other traditional risk factors ^[2]^. However, CRF is not measured or tracked for change during workplace wellness programmes, suggesting that employers are not completely aware of the employee’s CV health. Employers therefore need to be made aware of the cardioprotective benefits of physical activity and the importance of including CRF measurement in workplace wellness programmes. This case study demonstrates the effectiveness of a supervised and monitored exercise intervention programme on CRF, CV health and medical health claims of a high-risk employee of a corporate company.

## Case report

### History

A 49-year-old male employee presented with hypertension, obesity and depression. Table 1 outlines the baseline data of the participant. He was on anti-hypertensive and anti-depression medications and had been sedentary (not engaged in moderate-vigorous intensity physical activity for at least three days/week for three months). He decided to join an exercise facility at work to improve his health.

### Physical examination

An initial assessment included pre-participation exercise safety screening and the determination of CV risk factors using the diagnostic criteria published by the American College of Sports Medicine (ACSM) ^[3]^. Height (cm) and mass (kg) were measured using the Detecto stadiometer (USA) and Tanita Body Composition Scale (BF-350, Tokyo) respectively. The body mass index (BMI) was calculated in kg.m^2^. Blood pressure (mmHg) was measured three times, consecutively, in a seated, rested position with a Rossmax International (Taiwan) electronic blood pressure cuff, using a standardised method. The average of the two latter measures was used. Waist circumference (cm) was measured at the greatest abdominal circumference between the lowest rib and the iliac crests. Point-of-care fasting, full lipogram and blood glucose tests were conducted using standardised protocols.

CRF was measured as VO_2_peak using the Technogym® (TG) submaximal test on a TG treadmill. This test estimates maximal oxygen consumption (VO_2_peak) using the linear relationship between heart rate and VO_2_ max without subjecting the individual to high levels of physical stress. During the TG submaximal exercise testing, predetermined workloads are used to elicit a steady state of exertion (plateau in heart rate and VO_2_). The steady state heart rate at each workload is then calculated and extrapolated to the VO_2_ at the age-predicted maximal heart rate. This test is a double stage submaximal test and its validity and reliability has been documented ^[4]^.

Medical health claims were provided by the participant for three months before, during and after the exercise intervention programme.

### Intervention

The exercise intervention programme was administered in the exercise facility and was part of a corporate wellness programme at the participant’s workplace. The participant was assigned an individualised 12-week exercise programme, designed on the MyWellness® Technogym® Cloud Platform, and based on the American College of Sports Medicine’s (ACSM) guidelines for exercise testing and prescription regarding hypertension ^[3]^. The participant could access the programme on the mobile application of the MyWellness® Technogym® Cloud. The online cloud platform allows for real-time exercise data to be collected from the exercise equipment and third-party connected devices. Thus all exercise/physical activity data (in and outside the exercise facility) of the participant were collected during the intervention period. A biokineticist (exercise rehabilitation specialist) supervised these individual exercise sessions and monitored any physiological changes using the TG MyWellness® cloud. The exercise programme was adjusted according to the participant’s blood pressure, heart rate and adaptation to the exercise routine. Each contact session was also used to motivate the participant. The assigned weekly exercise volume was used as the intervention’s primary goal. The biokineticist provided verbal motivation to the participant on achieving this goal and attending the sessions. After the 12-week post-assessment, the biokineticist discussed the results and health outcomes with the participant.

### Results and outcomes

The measurements of resting heart rate, waist circumference, systolic and diastolic blood pressures, and peak VO_2_ improved at follow-up (Table 1). Weight and BMI increased slightly. Triglycerides, low-density lipoprotein cholesterol, and high-density lipoprotein cholesterol improved post-intervention, while total cholesterol and blood glucose increased slightly and remained within healthy ranges. The number of CV risk factors decreased from six at baseline to four at follow-up. It should be noted that of the six modifiable risk factors (smoking, inactivity, hypertension, dyslipidaemia, obesity, prediabetes), the number halved from four to two.

**Fig. 1:**
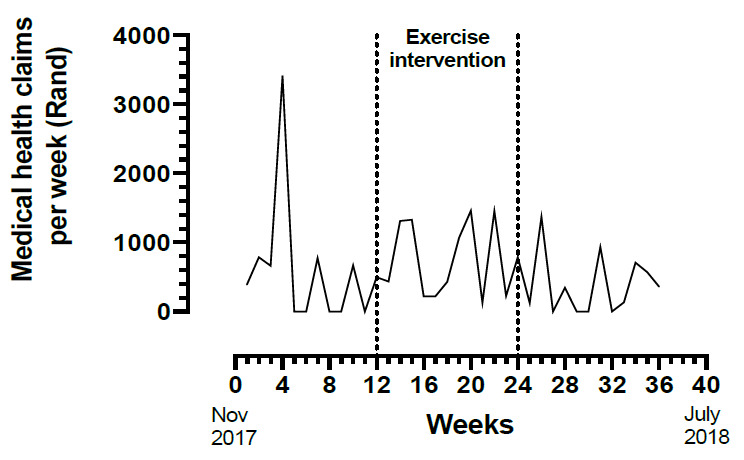
Medical health claims per week. The data are presented from November, Week One 2017 to July, Week One 2018. The exercise intervention took place between Weeks 12 and 24.

Total medical health claims for the three months pre-intervention equalled R7 199 (ZAR). This value went up to R9 076 during the intervention and then was reduced to R4 558, three-months post-intervention (Fig. 1). The total medical health costs were reduced by 37% from pre-intervention to three-months post-intervention. A more comprehensive account of medical healthcare costs was not provided.

## Discussion

The exercise intervention programme improved the health outcomes of this participant and reduced the number of CV risk factors (Table 1). Weight and BMI did not improve; however, waist circumference decreased slightly. The improvement in high-density lipoprotein cholesterol is encouraging as this is a negative CV risk factor that is beneficial to CV health. The greatest change occurred with CRF (VO_2_ peak) that improved by 68%, despite no weight loss.

The health outcomes of this case study agree with other published research of workplace wellness programmes ^[1]^. These studies though have not reported on changes in CRF.

CRF has been identified as an independent predictor of CV and all-cause mortality. This is also the risk factor that attributes the highest percentage to all-cause deaths when compared to other traditional risk factors ^[2]^. Furthermore, the American Heart Association recently proposed a case for CRF as a clinical vital sign ^[5]^. Therefore, workplace wellness programmes should place an importance on measuring, tracking and improving CRF via exercise interventions. As previously mentioned, in this case study there was an improvement in CRF following supervised exercise intervention.

There is also a lack in research accurately reporting on the specifics of the exercise prescription used in exercise interventions (e.g. exercise intensity or weekly volume) within corporate wellness programmes and especially for the treatment and prevention of chronic diseases. This case study focused on monitoring and recording the participant’s exercise and physical activity sessions. The participant expended an average weekly exercise volume of 19 kcal.kg^−1^week^−1^ during the intervention phase. This volume agrees with the recommended dose from the current physical activity guidelines. Ding et al. ^[6]^ also showed the importance of targeting sedentary behaviour within the spectrum of lifestyle risk behaviours in reducing all-cause mortality. CRF is a simple method for monitoring the transition from a state of being sedentary to becoming completely aligned with the target of 150 moderate-vigorous physical activity minutes per week for the prevention of CV disease.

An interesting finding in this case-study is that the medical health claims increased during the intervention period and reduced in the post-intervention period to levels below those of the pre-intervention period. This finding could be confounded by other factors, such as change in medical claim behaviour during the fiscal year, or expenses unrelated to the presenting chronic condition and would require further investigation. Nevertheless, the data suggest a spillover effect of exercise to lower healthcare claims following supervised exercise. In addition, the participant decided to continue being physically active despite exiting the supervised exercise intervention- (data on the MyWellness® Technogym® Cloud platform confirmed this). The intervention programme may have played a role in motivating the employee to continue being physically active. The motivational aspect of an exercise intervention programme is noteworthy.

A limitation of the case study is the restricted data on specific healthcare claims and the pattern of claims prior to entry into the exercise intervention programme. Further research is needed using randomised control trials to determine the longitudinal patterns of healthcare claims and other outcomes, such as illness-related absenteeism, in employed individuals with single and multiple chronic conditions.

## Conclusion

This exercise intervention programme had beneficial effects on the CRF, health and medical health claims profile of the employee. The greatest change occurred in CRF, supporting the need for including the assessment and monitoring of this factor in corporate wellness programmes.

## Figures and Tables

**Table 1:** Changes in physical characteristics and physiological and cardiometabolic parameters

Parameters	Baseline	Follow-up	% change	Absolute change
Age (yrs)	49	49	0	0
Resting heart rate (bt.min^−1^)	85	78	−8	−7
Height (cm)	174	174	0	0
Weight (kg)	99	100	1	+0.8
BMI (kg.m^2^)	32.7	33	1	+0.3
Waist circumference (cm)	111	110	−1	−1
Systolic blood pressure (mmHg)	149	139	−7	−10
Diastolic blood pressure (mmHg)	108	99	−8	−9
Total cholesterol (mmol.L^−1^)	2.59	2.73	5	+0.14
Low Density Lipoproteins (mmol.L^−1^)	1.05	0.98	−7	−0.07
Triglycerides (mmol.L^−1^)	3.31	2.18	−34	−1.13
High Density Lipoproteins (mmol.L^−1^)	0.88	1	14	+0.12
Glucose (mmol.L^−1^)	4.3	4.5	5	+0.2
Peak VO_2_ (ml.kg^−1^min^−1^)	25.2	42.3	68	+17.1
Weekly physical activity level (kcal.kg^−1^wk^−1^)	0	19.4	n/a	+19.4
**Cardiovascular (CV) risk factors** *				
Age	1	1++		
Family	1	1++		
Smoking	0	0++		
Inactivity	1	0†		
Hypertension	1	1++		
Dyslipidaemia	1	0†		
Obesity	1	1++		
Prediabetes	0	0++		
Total number of CV risk factors	6	4		

* 0, CV risk factor absent;

* 1, CV risk factor present;

† CV risk factor improved;

++ CV risk factor did not change.

BMI= weight (kg)/height^2^ (m)

## References

[1] Lutz N Taeymans J Ballmer C Cost-effectiveness and cost-benefit of worksite health promotion programmes in Europe: a systematic review Eur J Public Health 2019 29 3 540 546 10.1093/eurpub/cky269 30608540

[2] Myers J McAuley P Lavie CJ Physical activity and cardiorespiratory fitness as major markers of cardiovascular risk: their independent and interwoven importance to health status Prog Cardiovasc Dis 2014 57 4 306 314 10.1016/j.pcad.2014.09.011 25269064

[3] ACSM’s Guidelines for Exercise Testing and Prescription 10th ed 2016 Philadelphia Wouters Kluwer Chapter 2; 48, Chapter 10

[4] Cecchinelli F Senni S Paolo B Physical assessment with Technogym Fitness Equipment. Technogym Medical-Scientific Research Department. The 15th Sports Medicine Balkan Congress Medicina Sportiva 2008 14

[5] Ross R Blair SN Arena R Importance of assessing cardiorespiratory fitness in clinical practice: a case for fitness as a clinical vital sign. A scientific statement from the American Heart Association Circulation 2016 134 24 e653 e699 10.1161/CIR.0000000000000461 27881567

[6] Ding D Rogers K van der Ploeg H Traditional and emerging lifestyle risk behaviors and all-cause mortality in middle-aged and older adults: evidence from a large population based Australian cohort PLoS Med 2015 12 12 e1001917 10.1371/journal.pmed.1001917 26645683 PMC4672919

